# Modulation of Gene Expression by Polymer Nanocapsule Delivery of DNA Cassettes Encoding Small RNAs

**DOI:** 10.1371/journal.pone.0127986

**Published:** 2015-06-02

**Authors:** Ming Yan, Jing Wen, Min Liang, Yunfeng Lu, Masakazu Kamata, Irvin S. Y. Chen

**Affiliations:** 1 Department of Microbiology, Immunology, and Molecular Genetics, David Geffen School of Medicine at University of California Los Angeles, Los Angeles, California, United States of America; 2 Department of Biomolecular and Chemical Engineering, University of California Los Angeles, Los Angeles, California, United States of America; 3 California NanoSystems Institute (CNSI), University of California Los Angeles, Los Angeles, California, United States of America; 4 Division of Hematology-Oncology, David Geffen School of Medicine at University of California Los Angeles, Los Angeles, California, United States of America; 5 University of California Los Angeles AIDS Institute, Los Angeles, California, United States of America; The University of Tennessee Health Science Center, UNITED STATES

## Abstract

Small RNAs, including siRNAs, gRNAs and miRNAs, modulate gene expression and serve as potential therapies for human diseases. Delivery to target cells remains the fundamental limitation for use of these RNAs in humans. To address this challenge, we have developed a nanocapsule delivery technology that encapsulates small DNA molecules encoding RNAs into a small (30nm) polymer nanocapsule. For proof of concept, we transduced DNA expression cassettes for three small RNAs. In one application, the DNA cassette encodes an shRNA transcriptional unit that downregulates CCR5 and protects from HIV-1 infection. The DNA cassette nanocapsules were further engineered for timed release of the DNA cargo for prolonged knockdown of CCR5. Secondly, the nanocapsules provide an efficient means for delivery of gRNAs in the CRISPR/Cas9 system to mutate integrated HIV-1. Finally, delivery of microRNA-125b to mobilized human CD34+ cells enhances survival and expansion of the CD34+ cells in culture.

## Introduction

The recent explosion of small RNAs such as double-stranded siRNAs, gRNAs and miRNAs that regulate gene expression or directly edit genes has led to the potential for therapies based upon those RNAs[1–5]. The major advantages for their use are ease of design and test for activity. However, despite the wide applications to control gene expression in cells, effective delivery remains a major roadblock for future applications of these RNAs. Viral vectors allow endogenous synthesis of small RNAs and are able to provide sustainable gene regulation for primary human cells; however, cost and safety consideration constrains wide application[6–8]. Non-viral delivery systems such as electroporation and cationic chemical agents are popular choices for delivery of small RNAs in the form of DNA expression plasmids or *in-vitro* synthetic RNAs[9–12]. Unmodified synthetic RNAs are quickly degraded during delivery; relatively high doses are needed to achieve desired levels of gene modulation. Chemical modification is an effective means to increase the stability of small RNAs, however, extensive screening is required and may sometimes lead to undesired effects. Cationic chemical agents, such as lipofectamine, lipid-like materials, cell-penetration peptide (CPP) and cationic polymers, are widely used for *in vitro* studies of synthetic RNAs and showed potential for *in vivo* gene silencing, especially in the liver[5–8]; however toxicity and limitations in delivery efficiency have limited their effectiveness. DNA plasmids that express encoded small RNAs are effective at lower concentrations than synthetic RNAs and generally last longer. However, DNA expression plasmids are much bigger than their corresponding RNAs and have reduced delivery efficiency when electroporation was used[13,14]. Smaller expression constructs for siRNA constructed by PCR provide an alternative to plasmids, but delivery requires further development[15]. Therefore, in spite of intensive efforts, the design and synthesis of an effective delivery vehicle for small RNAs remains a challenge.

## Materials and Methods

### Ethics Statement

All studies described in this manuscript have approval by the UCLA Institutional Review Board (IRB).

### Cells

293T cells (American Type Culture Collection (ATCC), Manassas, VA) were cultured in Dulbecco’s modified Eagle medium (DMEM) supplemented with 10% fetal bovine serum (FBS) and 100U/ml of penicillin and 100 μg/ml of streptomycin. 293-Affinofile cells were maintained in DMEM supplemented with 10% dialyzed FBS (12–14 kD dialyzed; Atlanta Biologicals) and 50μg/ml blasticidin (D10F/B). Affinofile cell line was generously provided by Dr. Benhur Lee. CEM T clone cell lines were obtained from Calimmune, Inc. The clones were transfected with FG11 GFP vectors at MOI 0.01 to achieve one vector copy per cell. CEM T clones (Clone 1 and 2) were maintained in RPMI 1640 medium (Life Technologies, Carlsbad, CA) containing 10% FBS and supplemented with 2mM glutamine, 100 U/ml of penicillin, and 100 μg/ml of streptomycin. Mobilized human CD34+ hematopoietic stem cells (AllCells, Alameda, CA) were cultured in StepSpan SFEM medium (Stemcell Technologies, Tukwila, WA) with 1% Antibiotic-antimycotic (Gibco, by Life Technologies, Carlsbad, CA), 1% glutamine, 50ng/mL SCF, 50ng/mL TPO and 50ng/mL Flt-3 ligand.

### DNA cassettes

The CCR5-shRNA DNA cassette was amplified by PCR from sh1005 pBluescript plasmid with T3 and T7 primers from 2843bp to 47bp. The sequence of DNA cassette is listed in Supporting Information. The DNA cassette encoding 266bp U6 promoter and 103bp gRNA was synthesized by IDT gBlock and further amplified by PCR with T3 and T7 primers. The target sequence of gRNA1[16] and gRNA2 were TTAGACCAGATCTGAGCCT and CAGAGAGACCCAGTACAGTC, respectively. The mi125b DNA cassette was amplified from miR-125b vector pVAX1 provided by David Baltimore[17]. The sequence of primers are 5’-TGGACTAGTCGTGAGGCTCCGGTGCCCGT-3’ and 5'-GCGAGATACCAGTGTACTAG-3', respectively.

### Synthesis of DNA cassette nanocapsules

The DNA PCR product was dissolved in 20uL Rnase-free water at 20uM. Then a specific amount of acryl-spermine, tris-acrylamide, degradable crosslinker (Glycerol 1,3-diglycerolate diacrylate, GDGDA) and non-degradable crosslinker (N,N’-methylenesbisacrylamide, BIS) dissolved in 0.5mL deoxygenated and deionized water was added to the 0.75mL microcentrifuge tube. Radical polymerization from the surface of the acryloylated protein was initiated by adding 0.02 mg of ammonium persulfate dissolved in 2 μL of deoxygenated and deionized water and 0.4 μL of N,N,N',N'-tetramethylethylenediamine. The reaction was allowed to proceed for 60 min in a nitrogen atmosphere.

### Characterization of DNA cassette nanocapsules

TEM images of nanocapsules were obtained on a Philips EM120 TEM at 100000X. Before observation, DNA cassette nanocapsules were negatively stained using 1% pH 7.0 phosphotungstic acid (PTA) solution. Zeta potential and particle size distribution were measured with a Malvern particle sizer Nano-ZS.

### *In vitro* intracellular delivery of the DNA cassette nanocapsules

Cellular internalization studies were assessed via fluorescence microscopic technique and fluorescence-activated cell sorting (FACS). HEK-293T cells and CWR cells were cultured in Dulbecco’s modified Eagle’s medium (DMEM) supplemented with 10% fetal calf serum (FCS) and 1% penicillin/streptomycin. Cells (5000 cells/well, 96-well plate) were seeded the day before adding the DNA nanocapsules. DNA nanocapsules with different concentrations were added into the cell medium. After incubation at 37°C for 2 to 4 hrs, the cells were washed three times with PBS and either visualized with Zeiss Axio Observer. Z1 fluorescence microscope or trypsinized, centrifuged, and re-suspended in 2% FCS/PBS and analyzed via FACS. CEM T cells were co-transfected with PEI-Cas9 and DNA cassette nanocapsules in Opti-MEM reduced serum media at 37°C for 4 hrs. After transfection, cells were washed by PBS and cultured in fresh RPMI media. The level of GFP expression was analyzed 5 days after transfection. Flow cytometry was performed with BD LSRFortessa X-20, and data were analyzed using Flowjo software.

### Sequence analysis of the gene editing by CRISPR/Cas9

Genomic DNA extracted from CEM T cell clone 1 by PCR with three primer sets, which were designed homologous to cell sequences flanking the integration site on the genome. For sequence analysis, the PCR product amplified with primer set 1 (sense: GTCCCAACTCATTTGGATTAC, antisense: GAAAAGGAAAGAGTCGTGTG) was cloned into pBluescript KS(-) vector and sequenced with M13 forward primer.

### Apoptosis induced by staurosporine

Mobilized CD34+ hematopoietic stem cells were maintained in StemSpan SFEM medium (Stemcell Technologies, Tukwila, WA) with 50ng/mL SCF, 50ng/mL TPO and 50ng/mL Flt-3 ligand. 2uM staurosporine was directly added into the culture medium and the cells treated for 4 hours. After treatment, cells were washed with SFEM medium twice and re-cultured in SFEM medium with 50ng/mL SCF, 50ng/mL TPO and 50ng/mL Flt-3 ligand for 24 hours for Annexin V staining and flow cytometry assays.

### Cas9/Cas9-D10A plasmids

The Cas9 nuclease plasmid (hCas9) was obtained from Addgene (Plasmid #41815), and the Cas9 D10A nickase plasmid (hCas9-D10A) was also obtained from Addgene (Plasmid #41815).

### Synthesis of PEI-condensed Cas9/Cas9-D10A plasmid

A low molecular weight polyethyleneimine (PEI 2 kDa) was modified with dioleoylphosphatidylethanolamine (PE) to form the PEI-PE conjugate. Simply, Succinyl PE, 1,2-dioleoyl-sn-glycero-3-phosphoethanolamine-N-(succinyl) (sodium salt) (Avanti Polar Lipids, INC.) was dissolved in DMSO for 10% stock solution and further diluted into the reaction concentration by Rnase-free water. 1-Ethyl-3-[3-dimethylaminopropyl]carbodimide hydrochloride (EDAC) and *N*-hydroxysuccinimide (NHS) were added to obtain NHS-activated-PE. NHS-activated-PE was added drop-wise into the PEI solution to form PEI-PE conjugate. The reaction was allowed to proceed overnight at room temperature. The product PEI-PE was purified by dialysis against Rnase-free water. The Cas9 or Cas9-D10A plasmid was condensed by PEI-PE at an N/P ratio of 16.

### 293-Affinofile cellular surface expression of CD4 and CCR5

CD4 and CCR5 receptor expression was induced with doxycycline (doxy; Invitrogen) and ponasterone A (ponA; Invitrogen), respectively, and induced as previously described[18]. Briefly, cells were induced with doxy (6 ng/ml) and ponA (5 uM) for 18–24 hours at 37uC and receptor expression was measured using cytometry following staining with either phycoerythin (PE)-conjugated anti-human CD4 antibody (clone Q4120, BD Biosciences) or PE-conjugated mouse anti-human CCR5 antibody (clone 2D7, BD Biosciences).

### Single-cycle infection of 293-Affinofile cells

Env-pseudotyped luciferase reporter viruses were first tittered in triplicate in a 96-well plate format on 293-Affinofile cells expressing the maximum induction levels for both CD4 (6 ng/ml doxy) and CCR5 (5 mM ponA) surface expression as previously described. In order to ensure that each infection assay was performed within the linear range, we used the volume of each virus needed to produce 800,000 relative light units (RLUs) when used to infect Affinofile cells expressing the highest levels of CD4 and CCR5. Cells (5x10^5^ cells/well, 6-well plate) were seeded the day before adding the DNA nanocapsules. DNA nanocapsules with different concentrations were added into the cell medium. After incubation at 37°C for 4 hrs, the cells were washed three times with PBS and cultured at the condition (6 ng/ml doxy and 5 mM ponA) for 3, 5 and 9 days. Two days prior to infection, 96-well, black tissue culture plates were coated with 10% poly-L-lysine and then seeded with 293-Affinofile cells transduced with DNA nanocapsules at a density of 10^4 cells/well. 18 to 24 hours later, the induction medium was removed and gently replaced with 100 ul of fresh, warmed culture medium containing env-pseudotyped virus. The infection plates were spinoculated at 2,000 rpm for 2 hours at 37°C, and then incubated for an additional 48 hours at 37°C. Infection medium was then removed, the cells were lysed, and luciferase activity was assayed using the luciferase assay system (Promega).

## Results and Discussion

### Nanocapsule delivery of DNA cassettes encoding shRNA

We recently reported a nanocapsule platform that efficiently encapsulates and delivers single small interfering RNAs directed to CCR5 (siRNA1005), the cellular chemokine receptor and HIV-1 co-receptor[19]. The well-characterized potency of siRNA1005 and therapeutic potential against HIV-1 made it an ideal candidate for demonstrating CCR5 knockdown by applying the single molecule nanocapsule delivery technology. We showed that CCR5 can be effectively knocked down by nanocapsules encapsulating siRNA1005. These nanocapsules protect the encapsulated siRNA from nucleases and can effectively deliver siRNA into cells. Here, we improved the technology to encapsulate a DNA molecule encoding a short hairpin RNA (shRNA) which is processed to form the siRNA.

**Fig 1:**
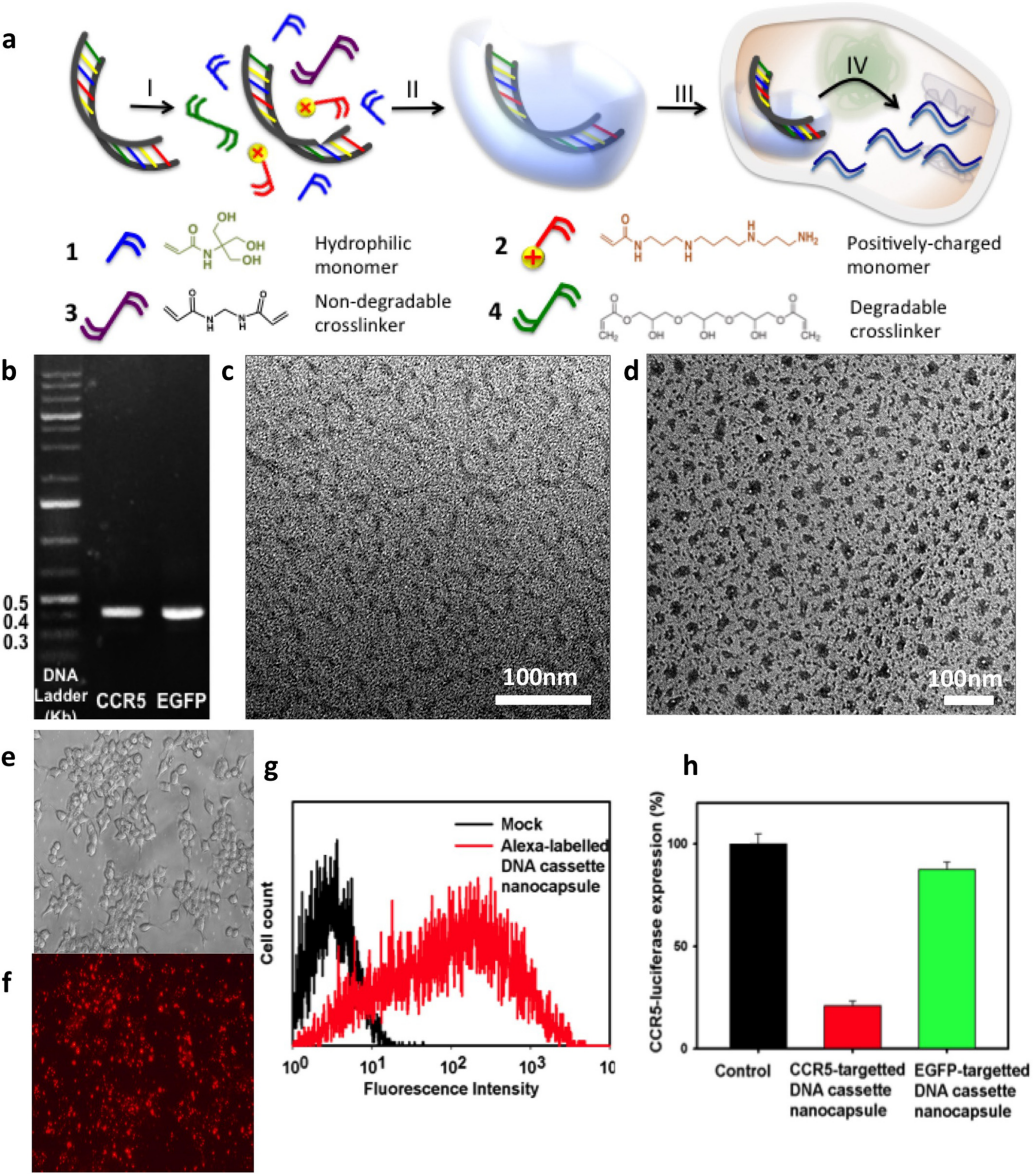
Preparation and characterization of DNA cassette nanocapsuels. a) Illustration of the synthesis and delivery of DNA cassette nanocapsules: 1) self-assembly of monomers, crosslinkers and DNA cassette; II): formation of DNA cassette nanocapsules through *in-situ* polymerization; III): delivery; IV): release of DNA cassette and expression of small RNAs. b) (A) Gel electrophoresis image of DNA cassette (a: for CCR5 shRNA; b: for EGFP shRNA). (B) TEM image of DNA cassettes (Scale bar = 100nm); (C) TEM image of DNA cassette nanocapsules (molar ratio of DNA to 3 reactants shown in Fig 1 as A,B,C = 1:750:750:30) c) HEK-293T cells transduced with Alexa592-labelled DNA cassette nanocapsules. (A) Optical image; (B) Fluorescence image; (C) Flow cytometry of 293T cells. D) Knockdown of CCR5-luciferase by CCR5-shRNA DNA cassette nanocapsule100ng DNA per 2.5 x104 cells in 100uL. Cells were dosed with Alexa592-labelled DNA cassette nanocapsules at 100nM for 4 h. Then nanocapsules were removed by washing 3 times with PBS. After trypsinization, cells were pictured with Leica Zeiss Axio Observer and also analyzed by a flow cytometer.

The fundamental principle in use of polymer nanocapsule technology is illustrated in Fig 1A. Starting with the hydrophilic and positively-charged monomers and crosslinkers, these molecules self-assemble along the surface of the DNA cassettes through electrostatic interaction and hydrogen bonding (Step **I**). Then, a thin network of polymer shell is formed around the DNA cassette by subsequent *in situ* polymerization (Step **II**), which effectively confers new surface properties and protects the DNA cassette. At a physiological pH of serum (pH~7.4), the nanocapsules are stable, but at lower pH in endosomes, there is escape into the cytoplasm due to the “proton-sponge” and membrane disruption effects (Step **III**), enabling release of the DNA cassette upon degradation of the shell. The DNA cassette then enters the nucleus where the small RNAs are expressed (Step **IV**).

Polymer nanocapsule technology to encapsulate encoding DNA rather than small RNAs is more challenging given the larger size of the DNA that includes not only the sequences encoding the RNAs, but also transcription promoters and flanking sequences. We designed a model DNA nanocapsule incorporating a short linear DNA cassette expressing sh1005 shRNA by an H1 pol III promoter[20–22]. The DNA cassette of only 395 base pairs (bp), produced by PCR from a plasmid construct (Fig 1B), has a much smaller size than the entire plasmid (3192 bp). We optimized the formulation of the polymer nanocapsule platform for encapsulation of the test DNA cassette by assaying various formulations for effectiveness in knockdown of a luciferase reporter gene expressed as a fusion mRNA of luciferase and CCR5[23]. We screened positively-charged monomers (Fig 1A & S1 Table), hydrophilic monomers (Fig 1A & S3 Table) and crosslinkers (Fig 1A & S2 Table) which alter the size, charge and degradability of the particles. We observed that a longer acid-degradable crosslinker (Glycerol 1,3-diglycerolate diacrylate) than that used for siRNA nanocapsules (1.3-glycerol dimethacrylate) was more effective for knockdown of CCR5-luciferase fusion mRNA by a DNA cassette encoding CCR5-specific shRNA.

The transmission electron microscope (TEM) image of linear naked DNA cassette stained with tungsten agent appears as a dark half circular arc with a diameter about 50 nm (Fig 1C); while the corresponding DNA cassette nanocapsules have a round morphology with a much smaller size around 30nm (Fig 1D), which is likely a result of DNA condensation through complexing with polymers. The DNA cassette nanoparticles are much smaller than the size of nanoparticles containing plasmid, around 150-300nm[10,12]. The small size of the DNA cassette nanocapsules favors a higher diffusion rate and likely improves delivery efficiency.

### Effective delivery of DNA cassette nanocapsules to cells

**Fig 2:**
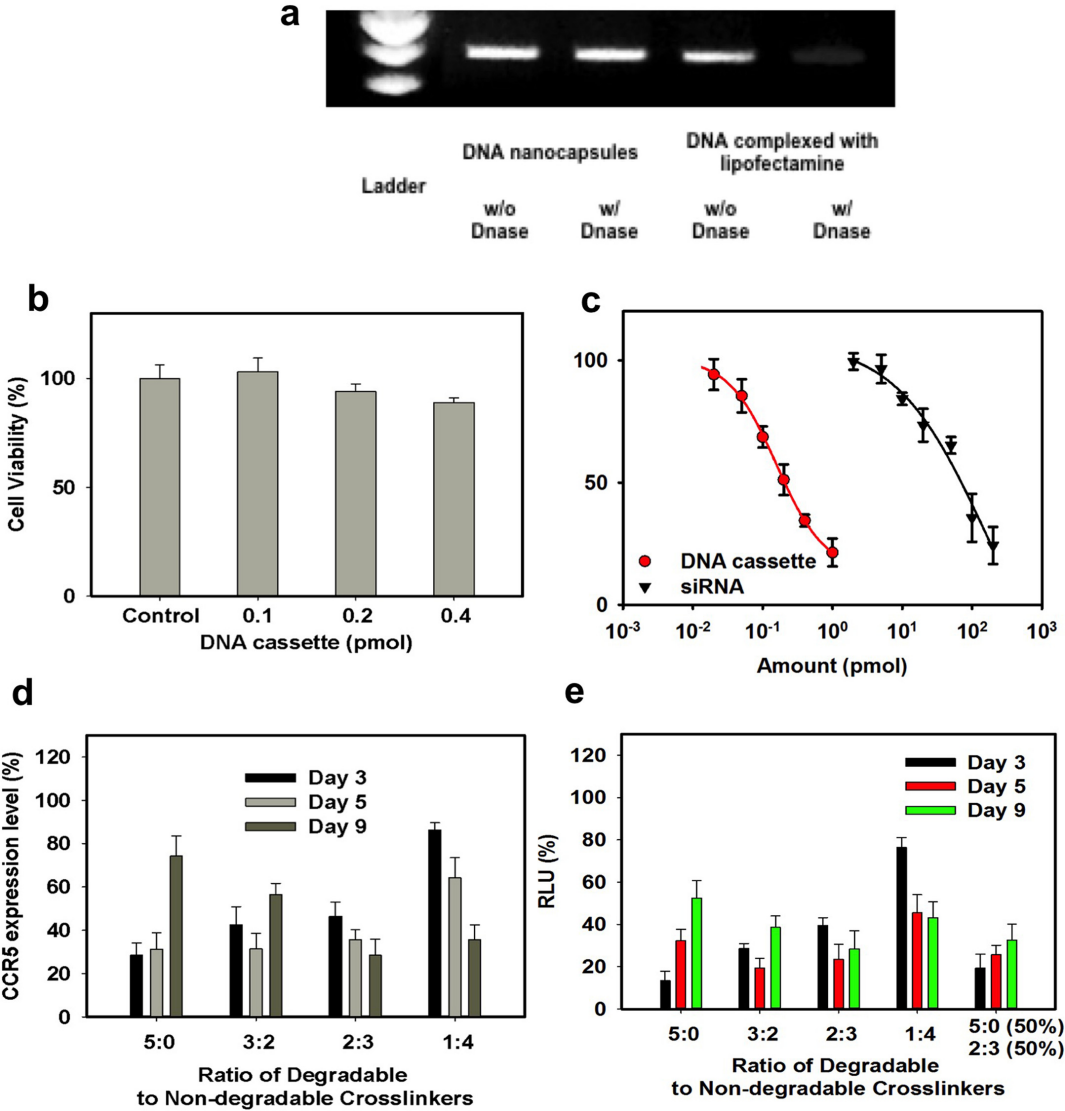
Nanocapsule delivery of shRNAs to downregulate CCR5 and inhibit HIV-1 infection. a) Sensitivity of DNA cassette to DNase I. DNA cassette complexed with lipofectamine and DNA nanocapsules were incubated for 1 hour without DNase I and with DNase I, respectively. b) Viability of HEK-293T cells transduced with CCR5 DNA cassette nanocapsules. HEK-293T cells were treated with DNA cassette nanocapsules at 0, 0.1, 0.2 and 0.4 pmol for 4 h at 37°C in 100uL of serum-free medium. After 24 h, cell viability was determined with CytoToxGlo kit using a 96-well plate reader. c) Knockdown of CCR5-Luciferase in HEK-293T cells by CCR5 DNA cassette nanocapsules and CCR5 siRNA lipofectamine complex. d) Down-regulation of CCR5 in 293 cells by sh1005 DNA cassette nanocapsules with different ratios (5:0; 3:2; 2:3 and 1:4) of degradable crosslinker (Glycerol 1,3-diglycerolate diacrylate, GDGDA) to non-degradable crosslinker (N,N’-methylenesbisacrylamide, BIS). On day 0, cells were transduced with DNA cassette nanocapsules for 4 hours. On day 3, 5 and 9, cells were stained and analyzed by flow cytometry. e) Inhibition of viral infection of Affinofile cells by sh1005 DNA cassette nanocapsules with different ratios (5:0; 3:2; 2:3, 1:4 and mixture of 5:0 (50%) and 2:3 (50%)) of degradable crosslinker (GDGDA) to non-degradable crosslinker (BIS). On day 0, Affinofile cells were transduced with DNA cassette nanocapsules for 4 hours and then cultured in the induction medium (6 ng/ml doxy and 5 mM ponA, respectively). On day 3, 5 and 9, Affinofile cells were seeded into 96-well plates at a density of 10^4 cells/well. 24 hours later, the induction medium was removed and gently replaced with 100ul of fresh, warmed culture medium containing env-pseudotyped virus. The infection plates were spinoculated at 2,000 rpm for 2 hours at 37°C, and then incubated for an additional 48 hours at 37°C. Infection medium was then removed, the cells were lysed, and luciferase activity was assayed using the plate reader.

The optical and fluorescent images of HEK-293T cells after incubation with Alexa592-labeled DNA cassette nanocapsules for 4 hrs is shown in Fig 1E. The intense red fluorescence demonstrates delivery of the Alexa592-labeled DNA nanocapsules (Fig 1F). Flow cytometry of HEK-293T cells transduced with Alexa592-labeled DNA cassette nanocapsules confirms the results of the fluorescence imaging and demonstrates successful delivery of fluorescence-labeled siRNA nanocapsule (Fig 1G). Transduction of 293T-cells by the CCR5-shRNA DNA nanocapsules downregulates about 80% of the bioluminescence intensity reflecting the knockout of the CCR5-luciferase fusion mRNA while cells treated with control shRNA EGFP DNA cassette nanocapsules did not exhibit significant decreases in the luciferase activity (Fig 1H). To investigate the sensitivity of DNA nanocapsules against Dnase I, DNA nanocapsules were incubated with Dnase I for 1 hour. After acid treatment and DNA extraction, agarose gel electrophoresis showed such nanocapsules could maintain the integrity of DNA (Fig 2A), while DNA is degraded in the native state or when formulated with a commercial lipofectamine reagent.The DNA nanocapsules did not show obvious cytotoxicity at the concentration of DNA cassette below 0.4 pmol. At 0.4 pmol, the viability of cells treated with DNA nanocapsules was slightly reduced to about 85% (Fig 2B).

We compared the knockdown efficacy of the DNA cassette nanocapsule with a standard lipofectamine siRNA transduction of HEK 293T cells expressing the CCR5-luciferase fusion gene (Fig 2C). On a molar basis, at 48 hours following transduction, the sh1005 DNA cassette nanocapsule is more than 200-fold more effective at downregulating CCR5 than siRNA1005 formulated with lipofectamine (Fig 2C), as expected due to de novo transcription of shRNA within nanocapsule transduced cells.

### Controlled release of DNA cassettes from nanocapsules

Considering the high potency of the DNA cassette nanocapsules, we reasoned that it could be used in applications where sustained activity is beneficial. The nanocapsules above are formulated with crosslinkers degraded under acidic conditions to allow release in endosomes. The DNA cassette nanocapsules were further engineered for controlled release by using a cocktail of acid-degradable and degradable non-acid crosslinkers[24]. The DNA nanocapsule prepared with 100% degradable crosslinkers (Glycerol 1,3-diglycerolate diacrylate, GDGDA) (S4 Fig) (5:0) was almost degraded completely after 2 hours at pH 5.4, while that prepared with the mixture of degradable crosslinker and non-degradable crosslinker (N,N’-methylene bisacrylamide, BIS) (S4 Fig) (1:4), degradation was completed after 4 hours (S4 Fig). In phosphate buffer solution at pH 7.4, these nanocapsules are quite stable at room temperature(S5 Fig) or 4°C(S6 Fig) for 2 weeks.

Crosslinkers with different ratios of degradable GDGDA and non-degradable BIS were included in the formulation of a nanocapsule carrying a luciferase-specific shRNA DNA cassette. Knockdown in cells was tested by using an HIV-1 bearing a luciferase reporter[18]. Our results show that the extent of HIV-1 (luciferase) downregulation is dependent upon the crosslinker ratio (Fig 2D). Nanocapsules with the highest percentage of degradable crosslinker (5:0) showed significant inhibition at the earliest point (Day 3), while the nanocapsules having increasing non-degradable crosslinker showed the best inhibition at later time-points. By mixing two nanocapsules with different ratios (5:0 and 2:3), HIV luciferase expression is maintained at a lower level throughout the 9 day test.

To demonstrate the effectiveness of sh1005 DNA nanocapsules in blocking HIV infection, the resistance of Affinofile cells[18] transduced with nanocapsules to HIV-env-pseudotyped luciferase reporter virus[18] was tested (Fig 2E). Sh1005 nanocapsules with 100% degradable crosslinker blocks 87%, 67% and 47% of infection of Affinofile cells at day 3, 5 and 9, respectively. Nanocapsules with 60%, 40% and 20% of degradable crosslinker blocked 81%, 76% and 54% of infection at day 5 and prevented 61%, 71% and 57% of infection at day 9, respectively. By mixing two nanocapsules with 100% and 40% of degradable crosslinker, respectively, 81%, 74% and 67% of infection was blocked at day 3, 5 and 9, respectively. Thus, the capability to release the DNA cassette intracellularly at controlled rate prolongs the inhibition of infection.

### Nanocapsule delivery of DNA cassettes encoding gRNAs to excise the HIV-1 provirus by CRISPR mutagenesis

**Fig 3:**
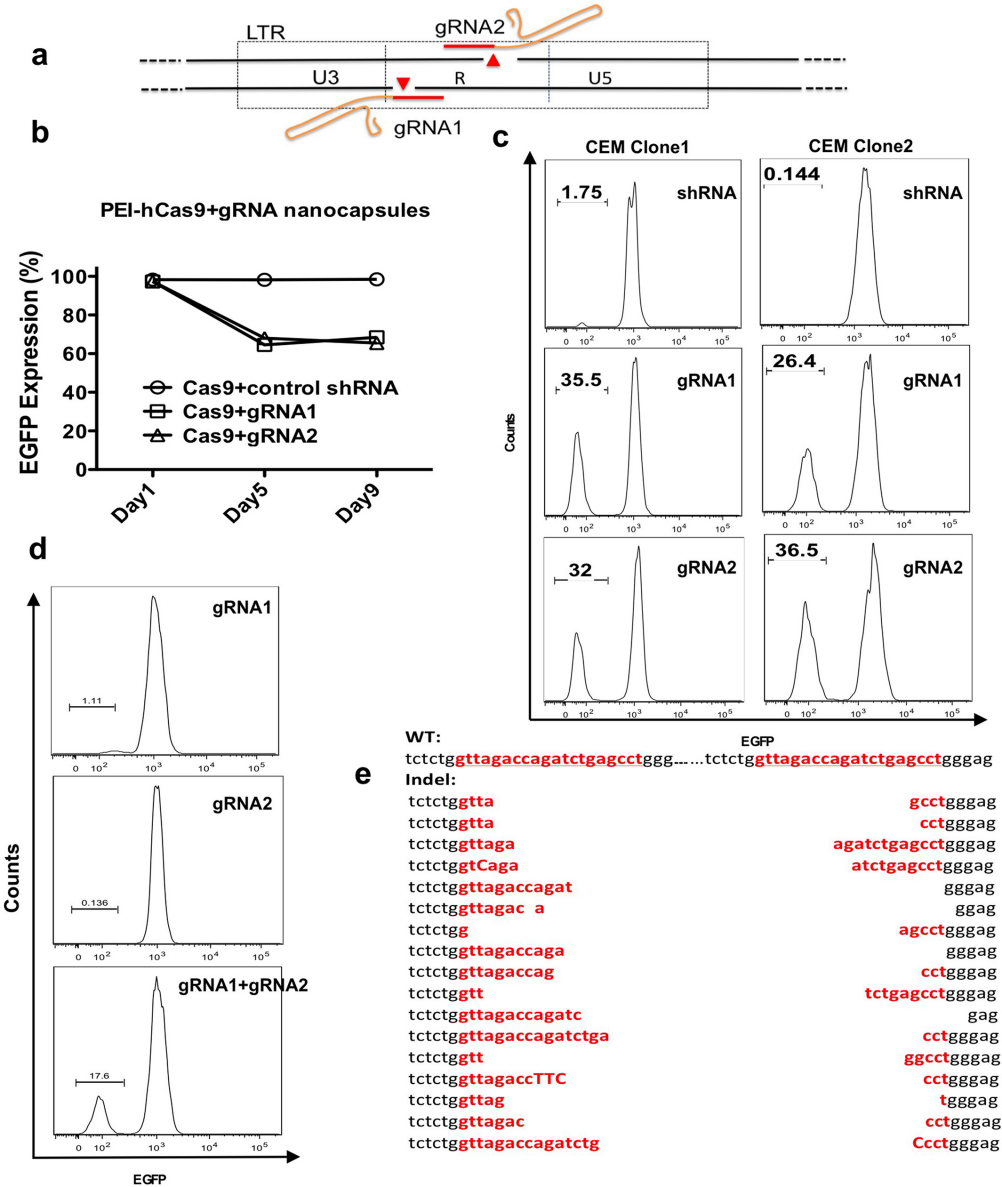
Nanocapsule delivery of gRNAs to excise the HIV-1 provirus by CRISPR mutagenesis. a) Schematic illustrating the position of two adjacent gRNAs directed to the HIV-1 LTR. b) A time course for knockout of EGFP expression was determined by flow cytometry. U6-control shRNA (CCR5-shRNA (a short linear DNA cassette with sh1005 shRNA expressed by U6 promoter)) was used a negative control. Dead cells were excluded by Live/Dead cell viability assay. c) Disruption of EGFP expression in two CEM-T4 clones, each bearing a single integrated EGFP lentiviral vector (FG11 EGFP). The U6-gRNA DNA cassette was encapsulated by nanocapsules, the hCas9 plasmid was condensed by PEI. The integration site of the lentiviral vector in Clone 1 is at chromosome (chr) 7 (-16350165). The integration site in Clone 2 is at chromosome (chr) 3 (+37026604). U6-control shRNA was used as a negative control. Dead cells were excluded by Live/Dead cell viability assay. Transduction efficiency is estimated to be 69.7% by transducing a Rhodamine B-labeled DNA cassette. d) EGFP expression after CRISPR/Cas9 nickase treatment. CEM-T4 cells were co-transduced with PEI condensed hCas9 nickase and gRNA nanocapsules. Dead cells were excluded by Live/Dead cell viability assay. e) Sequence analysis of the target site in the TAR region of LTR after the gRNA1/Cas9 treatment. DNA sequence demonstrated a single remaining LTR footprint resulting from proviral excision. The target sequence is indicated in red. The host cell genome sequence with integrated HIV vector is indicated as wild type (WT) on the top.

Targeted gene silencing using engineered nucleases has become a powerful method for biological research and a potential avenue for gene therapy[25]. Recently, a genome editing method using clustered, regularly interspaced, short palindromic repeat (CRISPR) technology, was identified in bacteria and archaea[26]. The advantage of this system over previous genome modifying activities is simplicity- a small guide RNA (gRNA) with homology to the target and a nuclease, Cas9, are all that is required to cleave specific target sequences[27,28]. However, effective delivery of CRISPR/Cas9 reagents into target cells is the key limitation for therapeutic applications[16,27]. Here, two gRNAs designed for mutagenesis of the HIV-1 genome, were used as model gRNAs (Fig 3A). By delivering these gRNAs with Cas9 into HIV-1 target cells, the provirus can be ablated. These DNA cassette nanocapsules were adapted for the delivery of a 409bp-DNA cassette encoding gRNA from a pol II U6 promoter, and the plasmid expressing Cas9 was transfected by dioleoylphosphatidylethanolamine conjugated branched polyethylenimine (PEI-PE). Our model system for evaluation of CRISPR/Cas9 gene editing consists of a CEM T-cell clone in which there is a single integrated HIV vector expressing EGFP from an internal Ubiquitin C (UbC) promoter. Unlike wild-type HIV provirus, where mutation of only the 5’ LTR would ablate transcription, this system provides a more stringent test for CRISPR/Cas9 activity in that loss of EGFP expression requires editing of both LTRs and excision of the intervening sequences. The kinetics of gene editing was determined by a time course for knockout of EGFP expression (Fig 3B). Knockout increased over time, 2 to 3% at Day 1 after transfection, stabilizing at about 30% by Day 5, and maintained to Day 9. One single transfection resulted in comparable knockout of around 30%, observed in two CEM clones with different vector integration sites (Fig 3C). Edited DNA fragments from CEM Clone 1 were isolated using primer sets flanking the provirus indicated in S7 Fig. By cloning the 750bp fragment, excised proviral fragments leaving a single LTR footprint were sequenced. 17 out of 20 clones contained various mutations at the predicted cleavage site (Fig 3E). The mutation patterns are consistent with non-homologous end joining (NHEJ) DNA repair observed after CRISPR/Cas9 generated double-strand DNA breaks[27].

Mutations of the catalytic residues (D10A in RuvC) of the Cas9 enzyme converts it into a DNA nickase[28]. As single-strand nicks are preferentially repaired by the high-fidelity base excision repair(BER) pathway, Cas9-nicking enzymes directed by a pair of gRNAs targeting opposite strands of a target locus mediated DSBs while minimizing off-target activity by 50 to 1500-fold[29,30]. By co-delivery of two gRNA nanocapsules, knockout of EGFP expression was 17% (Fig 3D).

### Nanocapsule delivery of DNA cassettes encoding miRNA to enhance survival and expansion of hematopoietic stem/progenitor cells (HSPC)

MicroRNAs (miRNAs) are major regulatory small RNAs and some miRNAs play important roles in development and differentiation of HSPCs [31]. Lentiviral vector-based expression of microRNA-125b (miR-125b) promotes the engraftment of cytokine mobilized CD34+ HSPC (mCD34+) cells and provides resistance to apoptosis; however, stable overexpression of miR-125b can induce abnormal phenotypes and leukemia[17,31–33]. One means to circumvent this problem is to transiently deliver miR-125b DNA cassettes to mCD34+ cells using nanocapsules.

**Fig 4:**
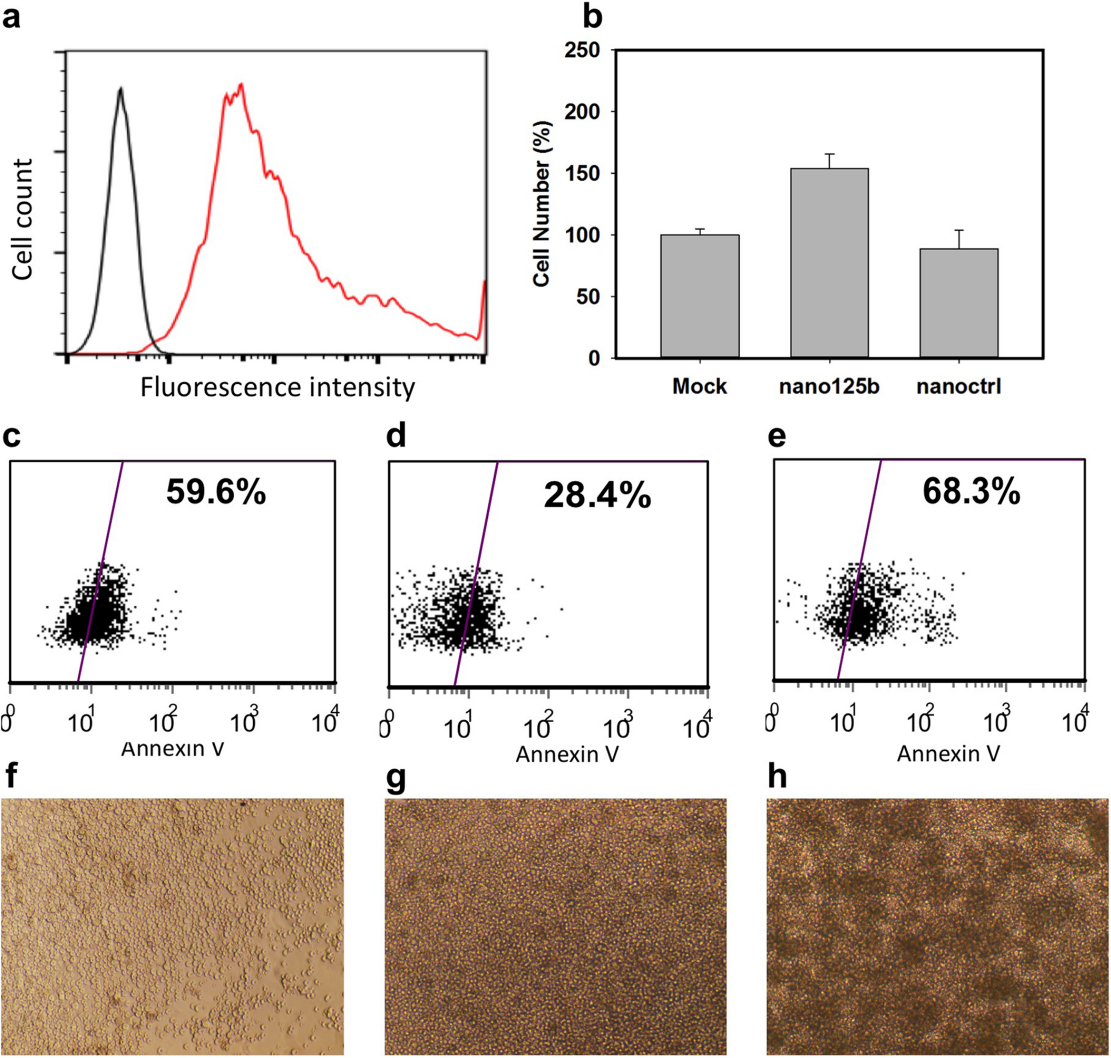
Nanocapsule delivery of miRNA to increase survival and expansion of hematopoietic stem/progenitor cells (HSPC). a) Flow cytometry of mobilized CD34+ cells incubated with fluorescence-labeled miR-125b DNA nanocapsules for 4 hours; b) *Ex-vivo* expansion of cytokine-mobilized CD34+ cells cultured with 50ng/mL SCF, 50ng/mL Flt-3L, and 50ng/mL TPO for 6 days after delivery of miR-125b DNA nanocapsules and control DNA nanocapsules. Annexin V staining of mobilized CD34+ cells c) without nanocapsules, d) with miR-125b DNA nanocapsules and e) with control DNA nanocapsules 24 hours later after treatment of staurosporine for 4 hours. Microscopic images of mobilized CD34+ cells f) without nanocapsules, g) with miR-125b DNA nanocapsules and h) transduced with miR-125b expressing lentiviral vector, cultured and expanded for 7 days.

A miR-125b DNA cassette (767 bps) consisting of the pol II EF1α promoter and miR-125b sequences was packaged in nanocapsules. Cytokine mobilized human CD34+ cells transduced with Alexa592-labeled DNA cassette nanocapsules show strong fluorescence in more than 99% of the population demonstrating effective delivery (Fig 4A). To confirm activity of miR-125b DNA nanocapsules, we demonstrated knockdown of luciferase expression following co-delivery of a plasmid expressing luciferase mRNA fused with the miR-125b target sequence (S8 Fig).

Following delivery of miR-125b DNA nanocapsules, mobilized CD34+ cells were expanded *ex vivo* with SCF, TPO and Flt-3 and monitored for 6 days. mCD34+ cells transduced by the miR-125b DNA cassette show a 60% gain in cell number compared with those without DNA cassette or with control DNA cassette (Fig 4B). To test the previously reported conferral by miR-125b of resistance to apoptosis, Annexin V staining of mobilized CD34+ cells was determined by flow cytometer 24 hours after treatment of 2uM staurosporine for 4 hours. The mCD34+ cells transduced with the miR-125b DNA cassette shows more than 50% reduction of apoptosis measured by Annexin V+ cells (Fig 4D) compared with control cells (Fig 4C) or control DNA cassette (Fig 4E). Microscopic images of mCD34+ cells transduced with miR-125b DNA cassette after *ex-vivo* expansion for 7 days show a generally normal morphology with some aggregation (Fig 4G) similar to control mCD34+ cells (Fig 4F). However, mCD34+ cells stably transduced with a lentiviral vector and constitutively expressing miR-125b showed an abnormal phenotype of more pronounced aggregation (Fig 4H). Thus, transient expression of miR-125b DNA cassettes in mCD34+ cells using nanocapsules provides resistance to apoptosis and increased cell number without the abnormal phenotype associated with constitutive expression.

## Conclusions

We demonstrate that small DNA molecules, termed DNA cassettes, that encode shRNAs, microRNAs, or gRNAs can be delivered by small polymer nanocapsules (~30nm) with high efficiency to cell lines and primary human hematopoietic stem cells. DNA cassettes encoding small RNAs are more potent than delivery of small RNAs themselves since each DNA molecule can transcribe many small RNAs intracellularly using engineered pol II or pol III promoters. Since the polymer shell protects the DNA cargo, chemical modification of the DNA or RNA is not required to maintain stability of activity. Furthermore, the DNA cassette nanocapsules can be engineered with diverse crosslinkers for controlled release of the DNA cargo for extended activity.

Most applications for delivery of small RNAs use *in-vitro* synthetic RNA delivered by electroporation and cationic agents where small RNA delivery is limited by RNA intracellular concentrations and a short half-life of the RNA. DNA delivery to express transgenes using encoded promoter elements is usually achieved with plasmids. Electroporation is limited by use of naked DNA sensitive to nucleases, with difficulties of scale-up and generally high toxicity[13,14]. Cationic agents generally show less efficient delivery with plasmids into primary human cells[9–12]^,^ due to high dosage required and reduced cell viability. DNA expression cassettes used in our delivery technology can elevate the intracellular concentration and duration of activity for small RNAs. Since minimal bacterial plasmid sequences are present on the DNA cassette, potential adverse immune responses to bacterial CpG motifs is mitigated[34].

While expression from genomically integrated lentiviral vectors is ideal for those applications where permanent transgene expression is desired, constitutive expression may be detrimental for some purposes such as that illustrated with miR-125b in HSPC[17,31–33]. This non-viral polymer nanocapsule delivery platform has fewer concerns for safety since the expression is transient in nature and, as we show, can be further regulated by use of formulations that provide controlled release.

In summary, the polymer nanocapsule technology provides a universal platform that can easily be adapted for delivery and efficient expression of any small RNA for both experimental and, in the future, potential therapeutic applications.

## Supporting Information

S1 Appendix Synthesis and NMR data of Some Positively Charged Monomers. (DOCX)

S2 Appendix Sequence of CCR5-shRNA DNA cassette. (DOCX)

S1 Table Positively Charged Monomers For DNA cassette Nanocapsules. (DOCX)

S2 Table Crosslinkers For DNA cassette Nanocapsules. (DOCX)

S3 Table Hydrophilic Monomers For DNA cassette Nanocapsules. (DOCX)

S1 Fig Comparison of knockdown of luciferase gene expression using luciferase shRNA DNA cassette nanocapsules with different positively charged monomers. (DOCX)

S2 Fig Comparison of knockdown of luciferase gene expression using sh1005 DNA nanocapsules with different crosslinkers. (DOCX)

S3 Fig Comparison of knockdown of luciferase gene expression using sh1005 DNA nanocapsules with different hydrophilic monomers. (DOCX)

S4 Fig Degradation of DNA cassettes nanocapsules at pH 5.4. (DOCX)

S5 Fig Stability of DNA cassette nanocapsules at pH 7.4. (DOCX)

S6 Fig Stability of DNA cassette nanocaspule at pH 7.4 for 2 weeks. (DOCX)

S7 Fig Sequence analysis of the target site in the TAR region of LTR after the gRNA1/Cas9 treatment. (DOCX)

S8 Fig Knockdown of miR-125b luciferase mRNA by miR-125b DNA nanocapsules or control DNA nanocapsules in 293T cells. (DOCX)

## References

[1] Kim D, Rossi J (2007) Strategies for silencing human disease using RNA interference. Nature Reviews Genetics 8: 173–184. 17304245 10.1038/nrg2006

[2] Davidson B, McCray PJ (2011) Current prospects for RNA interference-based therapies. Nature Reviews Genetics 12: 329–340. 10.1038/nrg2968 21499294 PMC7097665

[3] Cong L, Ran F, Cox D, Lin S, Barretto R, et al (2013) Multiplex genome engineering using CRISPR/Cas systems. Science 339: 819–823. 10.1126/science.1231143 23287718 PMC3795411

[4] Jinek M, East A, Cheng A, Lin S, Ma E, et al (2013) RNA-programmed genome editing in human cells. eLife 2: e00471 10.7554/eLife.00471 23386978 PMC3557905

[5] Wang J, Quake S (2014) RNA-guided endonuclease provides a therapeutic strategy to cure latent herpesviridae infection. Proc Natl Acad Sci USA 111: 13157–13162. 10.1073/pnas.1410785111 25157128 PMC4246930

[6] Li M, Bauer G, Michienzi A, Yee J, Lee N, et al (2003) Inhibition of HIV-1 infection by lentiviral vectors expressing Pol III-promoted anti-HIV RNAs. Molecular Therapy 8: 196–206. 12907142 10.1016/s1525-0016(03)00165-5

[7] Miyagishi M, Taira K (2002) U6 promoter driven siRNAs with four uridine 3' overhangs efficiently suppress targeted gene expression in mammalian cells. Nat Biotechnol2002May;20(5):497–500 20: 497–500. 11981564 10.1038/nbt0502-497

[8] Holkers M, Maggio I, Henriques S, Janssen J, Cathomen T, et al (2014) Adenoviral vector DNA for accurate genome editing with engineered nucleases. Nature Methods 11: 1051–1057. 10.1038/nmeth.3075 25152084

[9] Al-Dosari M, Gao X (2009) Nonviral gene delivery: principle, limitations, and recent progress. AAPS 11: 671–681. 10.1208/s12248-009-9143-y 19834816 PMC2782077

[10] Jones C, Chen C, Ravikrishnan A, Rane S, Pfeifer B (2013) Overcoming nonviral gene delivery barriers: perspective and future. Molecular Pharmaceutics 10: 4082–4098. 10.1021/mp400467x 24093932 PMC5232591

[11] Wang W, Li W, Ma N, Steinhoff G (2013) Non-viral gene delivery methods. Current Pharmaceutical Biotechnology 14: 46–60. 23437936

[12] Ding Q, Regan S, Xia Y, Oostrom L, Cowan C, et al (2013) Enhanced efficiency of human pluripotent stem cell genome editing through replacing TALENs with CRISPRs. Cell Stem Cell 12: 393–394. 10.1016/j.stem.2013.03.006 23561441 PMC3925309

[13] Rao D, Vorhies J, Senzer N, Nemunaitis J (2009) siRNA vs. shRNA: similarities and differences. Advanced Drug Delivery Reviews 61: 746–759. 10.1016/j.addr.2009.04.004 19389436

[14] Yin H, Kanasty R, Eltoukhy A, Vegas A, Dorkin J, et al (2014) Non-viral vectors for gene-based therapy. Nature Reviews Genetics 15: 541–555. 10.1038/nrg3763 25022906

[15] Castanotto D, Li H, Rossi J (2002) Functional siRNA expression from transfected PCR products. RNA 8: 1454–1460. 12458798 10.1017/s1355838202021362 PMC1370351

[16] Ebina H, Misawa N, Kanemura Y, Koyanagi Y (2013) Harnessing the CRISPR/Cas9 system to disrupt latent HIV-1 provirus. Scientific Reports 3: 2510 10.1038/srep02510 23974631 PMC3752613

[17] O'Connell R, Rao D, Chaudhuri A, Boldin M, Taganov K, et al (2008) Sustained expression of microRNA-155 in hematopoietic stem cells causes a myeloproliferative disorder. Journal of Experimental Medicine 205: 585–594. 10.1084/jem.20072108 18299402 PMC2275382

[18] Johnston S, Lobritz M, Nguyen S, Lassen K, Delair S, et al (2009) A quantitative affinity-profiling system that reveals distinct CD4/CCR5 usage patterns among human immunodeficiency virus type 1 and simian immunodeficiency virus strains. Journal of Virology 83: 11016–11026. 10.1128/JVI.01242-09 19692480 PMC2772777

[19] Yan M, Liang M, Wen J, Liu Y, Lu Y, et al (2012) Single siRNA Nanocapsules for enhanced RNAi delivery. Journal of the American Chemical Society 134: 13542–13545. 10.1021/ja304649a 22866878 PMC4318836

[20] An D, Donahue R, Kamata M, Poon B, Metzger M, et al (2007) Stable reduction of CCR5 by RNAi through hematopoietic stem cell transplant in non-human primates. Proc Natl Acad Sci U S A 104: 13110–13115. 17670939 10.1073/pnas.0705474104 PMC1941789

[21] Shimizu S, Hong P, Arumugam B, Pokomo L, Boyer J, et al (2010) A highly efficient short hairpin RNA potently down-regulates CCR5 expression in systemic lymphoid organs in the hu-BLT mouse model. Blood 115: 1534–1544. 10.1182/blood-2009-04-215855 20018916 PMC2830759

[22] Shimizu S, Kamata M, Kittipongdaja P, Chen K, Kim S, et al (2009) Characterization of a potent non-cytotoxic shRNA directed to the HIV-1 co-receptor CCR5. Genetic Vaccines and Therapy 7: 8 10.1186/1479-0556-7-8 19515239 PMC2701936

[23] Pang S, Pokomo L, Chen K, Kamata M, Mao S, et al (2014) High-Throughput Screening of Effective siRNAs Using Luciferase-Linked Chimeric mRNA. PLoS One 9: e96445 10.1371/journal.pone.0096445 24831610 PMC4022502

[24] Yan M, Du J, Gu Z, Liang M, Hu Y, et al (2010) A novel intracellular protein delivery platform based on single-protein nanocapsules. Nature Nanotechnology 5: 48–53. 10.1038/nnano.2009.341 19935648

[25] Schiffer J, Aubert M, Weber N, Mintzer E, Stone D, et al (2012) Targeted DNA mutagenesis for the cure of chronic viral infections. Journal of Virology 86: 8920–8936. 10.1128/JVI.00052-12 22718830 PMC3416169

[26] Sampson T, Saroj S, Llewellyn A, Tzeng Y, Weiss D (2013) A CRISPR/Cas system mediates bacterial innate immune evasion and virulence. Nature 497: 254–257. 10.1038/nature12048 23584588 PMC3651764

[27] Sander J, Joung J (2014) CRISPR-Cas systems for editing, regulating and targeting genomes. Nature Biotechnology 32: 347–355. 10.1038/nbt.2842 24584096 PMC4022601

[28] Mali P, Yang L, Esvelt K, Aach J, Guell M, et al (2013) RNA-guided human genome engineering via Cas9. Science 339: 823–826. 10.1126/science.1232033 23287722 PMC3712628

[29] Ran F, Hsu P, Lin C, Gootenberg J, Konermann S, et al (2013) Double nicking by RNA-guided CRISPR Cas9 for enhanced genome editing specificity. Cell 154: 1380–1389. 10.1016/j.cell.2013.08.021 23992846 PMC3856256

[30] Cho S, Kim S, Kim Y, Kweon J, Kim H, et al (2014) Analysis of off-target effects of CRISPR/Cas-derived RNA-guided endonucleases and nickases. Genome Research 24: 132–141. 10.1101/gr.162339.113 24253446 PMC3875854

[31] O'Connell R, Chaudhuri A, Rao D, Gibson W, Balazs A, et al (2010) MicroRNAs enriched in hematopoietic stem cells differentially regulate long-term hematopoietic output. Proc Natl Acad Sci USA 107: 14235–14240. 10.1073/pnas.1009798107 20660734 PMC2922591

[32] So A, Sookram R, Chaudhuri A, Minisandram A, Cheng D, et al (2014) Dual mechanisms by which miR-125b represses IRF4 to induce myeloid and B-cell leukemias. Blood 124: 1502–1512. 10.1182/blood-2014-02-553842 25006123 PMC4148772

[33] Bousquet M, Harris M, Zhou B, Lodish H (2010) MicroRNA miR-125b causes leukemia. Proc Natl Acad Sci USA 107: 21558–21563. 10.1073/pnas.1016611107 21118985 PMC3003065

[34] Klinman DM, Yamshchikov G, Ishigatsubo Y (1997) Contribution of CpG motifs to the immunogenicity of DNA vaccines. J Immunol 158: 3635–3639. 9103425

