# Supplementary material for: Modulation of Gene Expression by Polymer Nanocapsule Delivery of DNA Cassettes Encoding Small RNAs
Source: PLoS One. 2015 Jun 2;10(6):e0127986. doi: 10.1371/journal.pone.0127986 (PMC4452785; doi:10.1371/journal.pone.0127986)
Supplement: S1 Appendix — (DOCX) [file pone.0127986.s001.docx]

**S1 Appendix Synthesis and NMR data of Some Positively Charged Monomers**

**Synthesis of N-(3-((4-aminobutyl) amino) propyl) acrylamide / N-(2-((2-aminoethyl)(methyl) amino) ethyl) acrylamide / N-(piperazin-1-ylmethyl) acrylamide/ N-(2-(bis(2-aminoethyl) amino) ethyl) acrylamide.** The preparation of monomers was achieved by reacting amine-containing precursor (N-(3-aminopropyl) butane-1,4-diamine / N-methylpropane-1,3-diamine / piperazin-1-ylmethanamine / N,N’-bis(2-aminoethyl)ethane-1,2-diamine) with acrylic acid, hydroxysuccinimide ester (AHS). Briefly, amine-containing precursors and AHS were dissolved in chloroform at 0.5 mol/L, respectively. Then, AHS was added into each of the amine-containing precursors at the molar ratio of 1:1 gradually at room temperature under vigorous stirring. After overnight reaction, the mixture was filtered to remove by-products. The filtrate was then dried by rotary evaporation, followed by re-dispersing in water. After removal of insoluble substance, the solution was lyophilized. Finally, the product was purified by thin layer chromatography. The yield was from 37% to 63%.

**1H NMR for N-(3-((4-aminobutyl) amino) propyl) acrylamide** (400 MHz, D_2_O): 6.46 (m, 1H, CH_2_=CHCO), 5.65 (m, 2H, CH_2_=CHCO), 3.27 (m, 2H, CONH‐CH_2_), 2.78 (m, 10H, CH_2_‐NH‐CH_2_ and CH_2_‐NH_2_), 1.75 (m, 4H, NH‐CH_2_‐CH_2_), 1.23 (m, 4H, NH-CH_2_‐(CH_2_)_2_‐CH_2_-NH)

**1H NMR for N-(piperazin-1-ylmethyl) acrylamide** (400 MHz, D_2_O): 6.53 (m, 1H, CH_2_=CHCO), 5.69 (m, 2H, CH_2_=CHCO) , 3.91 (m, 2H, CONH‐CH_2_-N), 2.28 (m, 4H, CH_2_‐NH‐CH_2_), 2.75 (m, 4H, CH_2_-NH-CH_2_)

**1H NMR for N-(2-((2-aminoethyl)(methyl) amino) ethyl) acrylamide** (400 MHz, D_2_O): 6.39 (m, 1H, CH_2_=CHCO), 5.58 (m, 2H, CH_2_=CHCO), 3.18 (m, 2H, CONH‐CH_2_-N), 2.54 (m, 2H, CH_2_‐NH‐CH_3_), 3.22 (m, 3H, NH-CH_3_)

**1H NMR for N-(2-(bis(2-aminoethyl) amino) ethyl) acrylamide** (400 MHz, D_2_O): 6.41 (m, 1H, CH_2_=CHCO), 5.62 (m, 2H, CH_2_=CHCO), 3.14 (m, 2H, CONH‐CH_2_-N), 2.48 (m, 6H, N‐(CH_2_)_3_), 2.65 (m, 4H, CH_2_-NH2)2
