# Supplementary material for: Modulation of Gene Expression by Polymer Nanocapsule Delivery of DNA Cassettes Encoding Small RNAs
Source: PLoS One. 2015 Jun 2;10(6):e0127986. doi: 10.1371/journal.pone.0127986 (PMC4452785; doi:10.1371/journal.pone.0127986)
Supplement: S2 Appendix — (DOCX) [file pone.0127986.s002.docx]

**Sequence of CCR5-shRNA** **DNA cassette** The sequence of CCR5-shRNA DNA cassette is aattaaccctcactaaagggaacaaaagctggagctccaccgcggtggcggccgctctagaccatggaattcgaacgctgacgtcatcaacccgctccaaggaatcgcgggcccagtgtcactaggcgggaacacccagcgcgcgtgcgccctggcaggaagatggctgtgagggacaggggagtggcgccctgcaatatttgcatgtcgctatgtgttctgggaaatcaccataaacgtgaaatgtctttggatttgggaatcttataagttctgtatgagaccacggatccccgagcaagctcagtttacaccttgtccgacggtgtaaactgagcttgctctttttctcgagggggggcccggtacccaattcgccctatagtgagtcgtatta.
