# Supplementary material for: Modulation of Gene Expression by Polymer Nanocapsule Delivery of DNA Cassettes Encoding Small RNAs
Source: PLoS One. 2015 Jun 2;10(6):e0127986. doi: 10.1371/journal.pone.0127986 (PMC4452785; doi:10.1371/journal.pone.0127986)
Supplement: S1 Table — (DOCX) [file pone.0127986.s003.docx]

**S1 Table Positively Charged Monomers For DNA cassette Nanocapsules**

| Index | Name | Structure | Number of Protonable amines |
| --- | --- | --- | --- |
| *#1* | *N*-(3-((4-(3-aminopropyl amino) butyl) amino) propyl) acrylamide |  | 3 |
| *#2* | *N*-(3-(4-aminobutyl amino) propyl) acrylamide |  | 2 |
| *#3* | *N*-(2-((2-aminoethyl) methylamino) ethyl) acrylamide |  | 1 |
| *#4* | *N*-(piperazin-1-ylmethyl) acrylamide |  | 2 |
| *#5* | *N*-(2-(bis(2-aminoethyl) amino) ethyl) acrylamide |  | 3 |
| #6 | N-(3-Aminopropyl) methacrylamide hydrochloride |  | 1 |
| #7 | Dimethylamino ethyl methacrylate |  | 1 |
| #8 | (3-Acrylamidopropyl) trimethylammonium hydrochloride |  | 1 |
| #9 | 2-aminoethyl methacrylate |  | 1 |
