# Supplementary material for: Modulation of Gene Expression by Polymer Nanocapsule Delivery of DNA Cassettes Encoding Small RNAs
Source: PLoS One. 2015 Jun 2;10(6):e0127986. doi: 10.1371/journal.pone.0127986 (PMC4452785; doi:10.1371/journal.pone.0127986)
Supplement: S2 Table — (DOCX) [file pone.0127986.s004.docx]

**S2 Table** **Crosslinkers For DNA cassette Nanocapsules**

| Index | Name | Structure |
| --- | --- | --- |
| *#1* | 1,3-glycerol dimethacrylate |  |
| *#2* | Glycerol 1,3-diglycerolate diacrylate |  |
| *#3* | N,N'-bis(acryloyl)cystamine |  |
| *#4* | bis[2-(methacryloyloxy)ethyl] phosphate |  |
| *#5* | N,N'-Methylenebisacrylamide |  |
