# Supplementary material for: Modulation of Gene Expression by Polymer Nanocapsule Delivery of DNA Cassettes Encoding Small RNAs
Source: PLoS One. 2015 Jun 2;10(6):e0127986. doi: 10.1371/journal.pone.0127986 (PMC4452785; doi:10.1371/journal.pone.0127986)
Supplement: S3 Table — (DOCX) [file pone.0127986.s005.docx]

**S3 Table Hydrophilic Monomers For DNA cassette Nanocapsules**

| Index | Name | Structure |
| --- | --- | --- |
| *#1* | *N*-(1,3-dihydroxy-2-(hydroxymethyl) propan-2-yl) acrylamide |  |
| *#2* | acrylamide |  |
| *#3* | *N*-(hydroxymethyl)acrylamide |  |
| *#4* | 2-hydroxyethyl acrylate |  |
| *#5* | 2-hydroxyethyl methacrylate |  |
