# Supplementary material for: Modulation of Gene Expression by Polymer Nanocapsule Delivery of DNA Cassettes Encoding Small RNAs
Source: PLoS One. 2015 Jun 2;10(6):e0127986. doi: 10.1371/journal.pone.0127986 (PMC4452785; doi:10.1371/journal.pone.0127986)
Supplement: S1 Fig — (DOCX) [file pone.0127986.s006.docx]

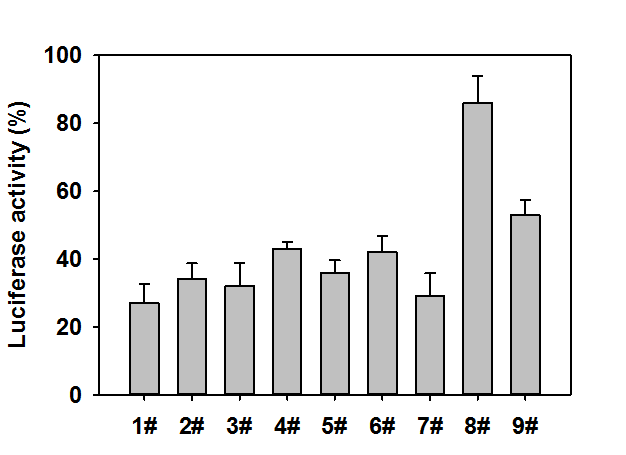


**S1 Fig.** **Comparison of knockdown of luciferase gene expression in luciferase-CCR5 expressing 293T cells using luciferase shRNA DNA cassette nanocapsules with different positively charged monomers** (#1-#14 in Table S1, #1 is also called acryl-spermine). Then a specific amount of positively charged monomer (Table 1), tris-acrylamide and glycerol dimethacrylate (total number of protonable amines of positively charged monomer : acrylamide : glycerol demthacrylate= 15:5:1) dissolved in 0.5mL deoxygenated and deionized water was added to the 0.75mL microcentrifuge tube. Radical polymerization was initiated by adding 0.02 mg of ammonium persulfate dissolved in 2 μL of deoxygenated and deionized water and 0.4 μL of N,N,N',N'-tetramethylethylenediamine. The reaction was allowed to proceed for 60 min in a nitrogen atmosphere. 293T cells were treated with DNA cassette nanocapsules at 50nM for 4 hours at 37 ^o^C in serum-free medium. The medium was changed to DMEM with 10% fetal bovine serum. After 48 hours, the luciferase activity was determined using a 96-well plate reader.
