# Supplementary material for: Modulation of Gene Expression by Polymer Nanocapsule Delivery of DNA Cassettes Encoding Small RNAs
Source: PLoS One. 2015 Jun 2;10(6):e0127986. doi: 10.1371/journal.pone.0127986 (PMC4452785; doi:10.1371/journal.pone.0127986)
Supplement: S2 Fig — (DOCX) [file pone.0127986.s007.docx]

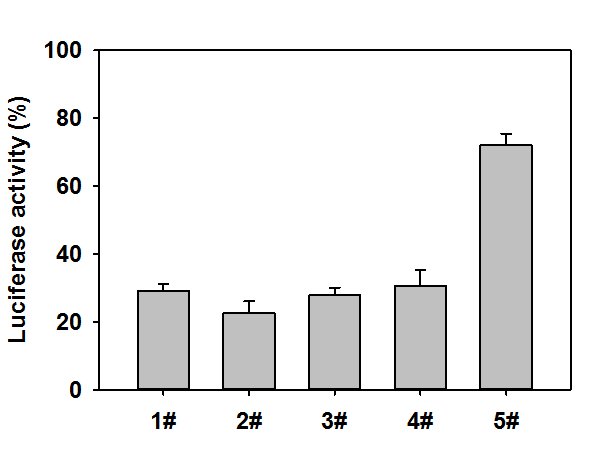


**S2 Fig.** **Comparison of knockdown of luciferase gene expression in luciferase-CCR5 293T cells using sh1005 DNA nanocapsules with different crosslinkers** (#1-#5 in Table S2). Then a specific amount of acryl-spermine, tris-acrylamide and crosslinker (#1-#5 in Table S2) (total number of protonable amines of positively charged monomer: tris-acrylamide: crosslinker = 15:5:1) dissolved in 0.5mL deoxygenate27d and deionized water was added. Radical polymerization was initiated by adding 0.02 mg of ammonium persulfate dissolved in 2μL of deoxygenated and deionized water and 0.4μL of N,N,N',N'-tetramethylethylenediamine. The reaction was allowed to proceed for 60 min in a nitrogen atmosphere. 293T cells were treated with DNA cassette nanocapsules at 0.5pmol for 4 hours at 37^o^C in serum-free medium. Then, medium were changed to DMEM with 10% fetal bovine serum. After 48 hours, the luciferase activity was determined using a 96-well plate reader.
