# Supplementary material for: Modulation of Gene Expression by Polymer Nanocapsule Delivery of DNA Cassettes Encoding Small RNAs
Source: PLoS One. 2015 Jun 2;10(6):e0127986. doi: 10.1371/journal.pone.0127986 (PMC4452785; doi:10.1371/journal.pone.0127986)
Supplement: S3 Fig — (DOCX) [file pone.0127986.s008.docx]

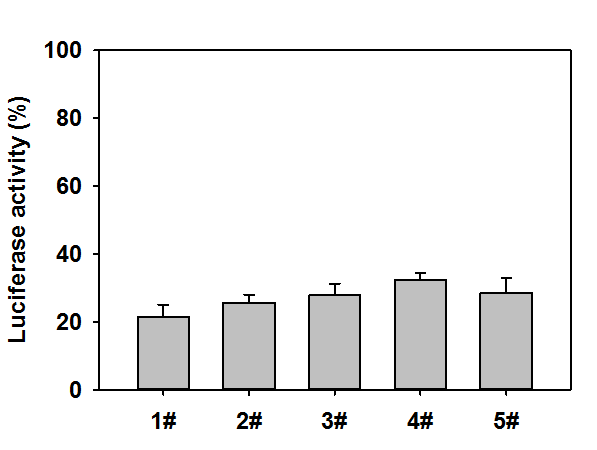


**S3** **Fig.** **Comparison of knockdown of luciferase gene expression in luciferase-CCR5 293T cells using sh1005 DNA nanocapsules with different hydrophilic monomers** (#1-#5 in Table S3). Then a specific amount of acryl-spermine, hydrophilic co-monomer (#1-#5 in Table S3) and glycerol 1,3-diglycerolate diacrylate (total number of protonable amines of acryl-spermine: neutral co-monomer : glycerol 1,3-diglycerolate diacrylate = 15:5:1) dissolved in 0.5mL deoxygenated and deionized water was added to the microcentrifuge tube. Radical polymerization from the surface of the acryloylated protein was initiated by adding 0.02 mg of ammonium persulfate dissolved in 2μL of deoxygenated and deionized water and 0.4μL of N,N,N',N'-tetramethylethylenediamine. 293T cells were treated with DNA cassette nanocapsules at 0.5pmol for 4 h at 37^o^C in serum-free medium. Then, medium were changed to DMEM with 10% fetal bovine serum. After 48 h, the luciferase activity was determined using a 96-well plate reader.
