# Supplementary material for: Modulation of Gene Expression by Polymer Nanocapsule Delivery of DNA Cassettes Encoding Small RNAs
Source: PLoS One. 2015 Jun 2;10(6):e0127986. doi: 10.1371/journal.pone.0127986 (PMC4452785; doi:10.1371/journal.pone.0127986)
Supplement: S4 Fig — (DOCX) [file pone.0127986.s009.docx]

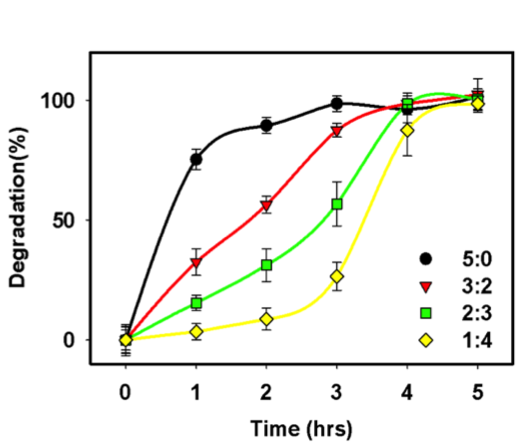


**S4 Fig. Degradation of DNA cassettes nanocapsules at pH 5.4 with cocktails of acid-degradable and non-acid-degradable crosslinkers (Ratio at 5:0, 3:2, 2:3 and 1:4).** The degradation degree was measured by dynamic light scattering and calculated as [R_0_–R_t_]/[R_0_–R*]X100%, where R_0_ is the initial diameter of DNA nanocapsules, R_t_ is the diameter of nanocapsules at time point, and R*is the diameter of a DNA molecule.
