# Supplementary material for: Modulation of Gene Expression by Polymer Nanocapsule Delivery of DNA Cassettes Encoding Small RNAs
Source: PLoS One. 2015 Jun 2;10(6):e0127986. doi: 10.1371/journal.pone.0127986 (PMC4452785; doi:10.1371/journal.pone.0127986)
Supplement: S6 Fig — (DOCX) [file pone.0127986.s011.docx]

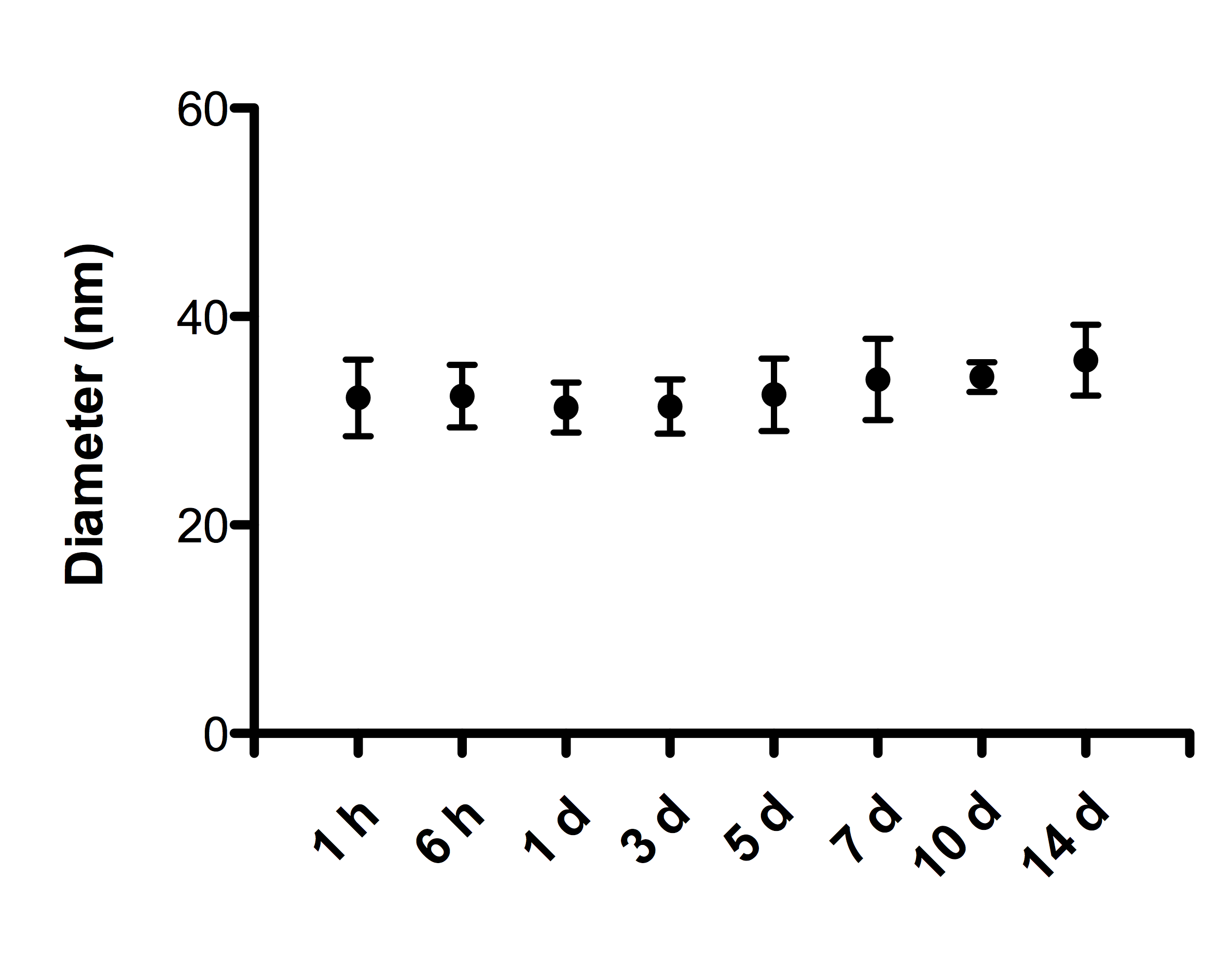


**S6 Fig. Stability of the DNA cassette nanocaspule at pH 7.4 for 2 weeks.** The DNA cassette nanocapsule was kept in PBS at 20nM at 4°C. The diameter of the nanocapsule was monitored by dynamic light scattering.
