# Supplementary material for: Modulation of Gene Expression by Polymer Nanocapsule Delivery of DNA Cassettes Encoding Small RNAs
Source: PLoS One. 2015 Jun 2;10(6):e0127986. doi: 10.1371/journal.pone.0127986 (PMC4452785; doi:10.1371/journal.pone.0127986)
Supplement: S7 Fig — (DOCX) [file pone.0127986.s012.docx]

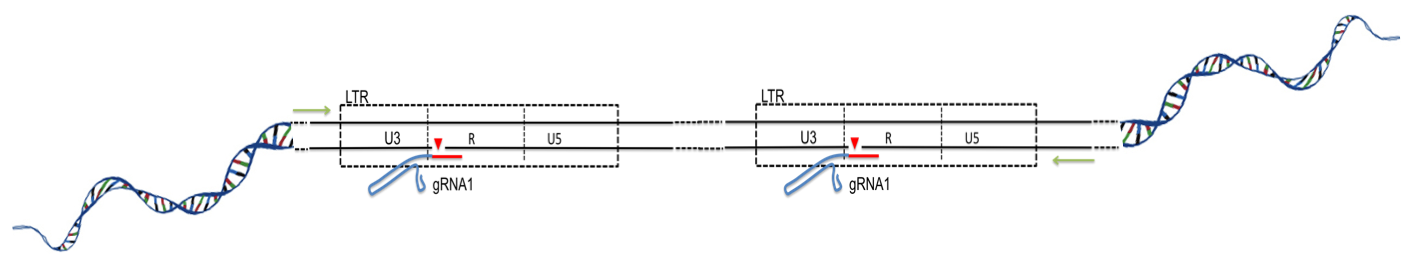
 **S7 Fig.**  **Sequence analysis of the target site in the TAR region of LTR after the gRNA1/Cas9 treatment.** The primer set used for CEM Clone 1 is shown as green arrows. Sense primer: GAAAAGGAAAGAGTCGTGTG. Antisense primer: GTCCCAACTCAT TTGGATTAC.
