# Supplementary material for: Modulation of Gene Expression by Polymer Nanocapsule Delivery of DNA Cassettes Encoding Small RNAs
Source: PLoS One. 2015 Jun 2;10(6):e0127986. doi: 10.1371/journal.pone.0127986 (PMC4452785; doi:10.1371/journal.pone.0127986)
Supplement: S8 Fig — (DOCX) [file pone.0127986.s013.docx]

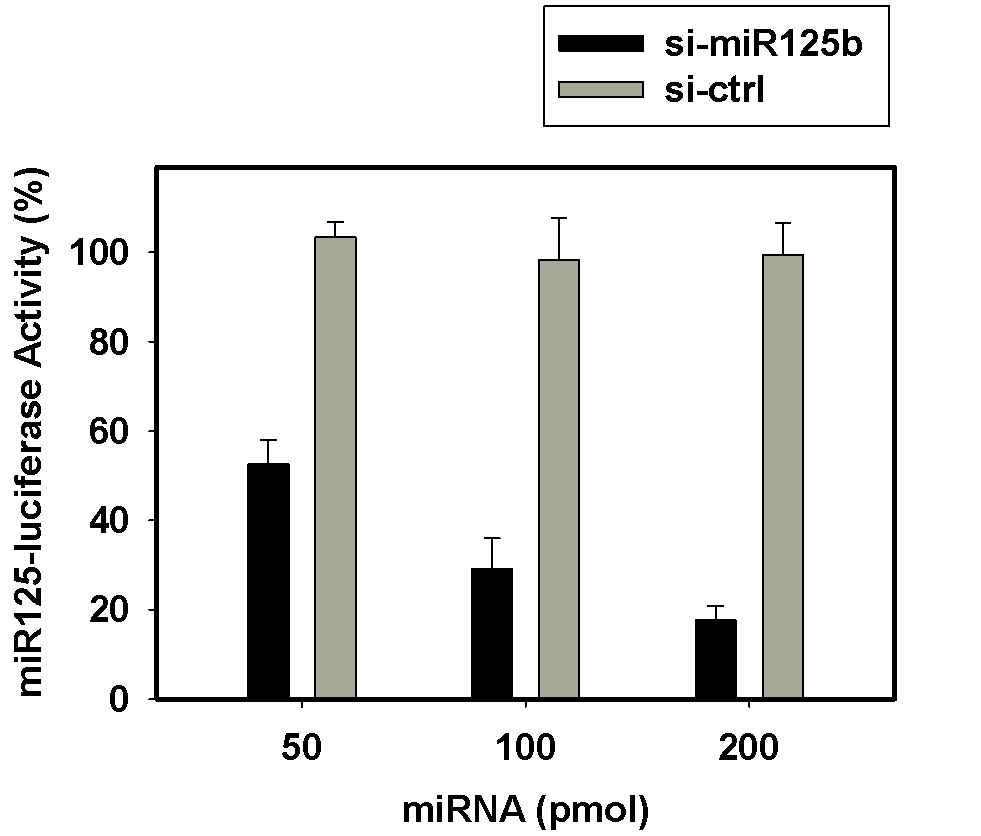


**S8 Fig. Knockdown of miR-125b luciferase mRNA by miR-125b DNA nanocapsules (Black bar) or control DNA nanocapsules (Grey bar) in 293T cells.** 293T cells were incubated with mixture of miR-125b luciferase plasmid and lipofectamine for 2 h then treated with miR125b DNA cassette nanocapsule or control DNA nanocapsules for 2 h at 37^o^C in serum-free medium. Then the medium was exchanged to fresh DMEM with 10% fetal bovine serum. After 48 h, the luciferase activity was determined using a 96-well plate reader.
